# Prediction of miRNA-Disease Association Using Deep Collaborative Filtering

**DOI:** 10.1155/2021/6652948

**Published:** 2021-02-24

**Authors:** Li Wang, Cheng Zhong

**Affiliations:** ^1^School of Computer Science and Engineering, South China University of Technology, Guangzhou 510006, China; ^2^School of Computer, Electronics and Information, Guangxi University, Nanning 530004, China

## Abstract

The existing studies have shown that miRNAs are related to human diseases by regulating gene expression. Identifying miRNA association with diseases will contribute to diagnosis, treatment, and prognosis of diseases. The experimental identification of miRNA-disease associations is time-consuming, tremendously expensive, and of high-failure rate. In recent years, many researchers predicted potential associations between miRNAs and diseases by computational approaches. In this paper, we proposed a novel method using deep collaborative filtering called DCFMDA to predict miRNA-disease potential associations. To improve prediction performance, we integrated neural network matrix factorization (NNMF) and multilayer perceptron (MLP) in a deep collaborative filtering framework. We utilized known miRNA-disease associations to capture miRNA-disease interaction features by NNMF and utilized miRNA similarity and disease similarity to extract miRNA feature vector and disease feature vector, respectively, by MLP. At last, we merged outputs of the NNMF and MLP to obtain the prediction matrix. The experimental results indicate that compared with other existing computational methods, our method can achieve the AUC of 0.9466 based on 10-fold cross-validation. In addition, case studies show that the DCFMDA can effectively predict candidate miRNAs for breast neoplasms, colon neoplasms, kidney neoplasms, leukemia, and lymphoma.

## 1. Introduction

miRNAMicroRNAs (miRNAs)areshort endogenous noncoding RNAs with about 22 nucleotides. A number of studies have shown that miRNAs play important roles in many biological processes including cell proliferation, development, differentiation, death, apoptosis, metabolism, aging, signal transduction, and viral infection [1–6]. Biological studies have revealed that dysregulation of miRNAs is closely related to the occurrence and development of complex diseases [7–9]. Dysregulation of miR-15 and miR-16 was discovered to be related with B-cell chronic lymphocytic leukemia firstly [10]. So far, it has been verified that many miRNAs are related to cancers. Five members of the miRNA-200 family (miR-200a, miR-200b, miR-200c, miR-141, and miR-429) are downregulated in the development of breast cancer [11]. Epigenetic modulation of the miR-200 family relates to transition to a breast cancer stem cell-like state [12]. Some studies demonstrate that in human colorectal cancer cells, miR-186, miR-216b, miR-337-3p, and miR-760 could work in synergy to induce cellular senescence by targeting the alpha subunit of protein kinase CKII [13]. By accurately measuring expression levels of miRNAs in the serum of 220 patients with early-stage non-small cell lung cancer and 220 matched controls, researchers found that the expressions of miR-27a, miR-106a, miR-221, miR-146b, miR-155, miR-17-5p, and let-7 were lower than those in controls, while the expression of miR-29c was increased [14].

Identifying the miRNAs associated with diseases will contribute to exploring the pathogenesis, diagnosis, treatment, and prognosis of diseases and help to develop new drugs. Some studies showed that miRNA-23, miRNA-24, and miRNA-27 contained underlying therapeutic factors in ischemic heart and vascular disease [15]. By targeting the BCL6 corepressor such as BCORL1, the migration and invasion of hepatocellular carcinoma (HCC) cells are restrained by miR-876-5p, which provides a new idea for the treatment of HCC [16]. However, the experimental methods for finding associations between miRNAs and diseases are expensive and time-consuming. The computational methods for predicting potential miRNA-disease associations can provide verifiable hypotheses for further experimental verification, which can reduce biological experiment time and improve the experimental efficiency.

Recently, plenty of computational methods have been proposed to predict potential miRNA-disease associations [17]. Most of the computational methods are based on the assumption that miRNAs with similar functions are more likely to be associated with phenotypically similar diseases and vice versa. These methods are based on different principles to predict miRNA-disease associations, such as similarity-based methods, machine learning-based methods, and matrix factorization-based methods.

The previous similarity-based computational methods were based on miRNA-target interaction network and protein-protein interaction (PPI). For example, Jiang et al. [18] proposed a method to predict potential miRNA-disease associations by applying a scoring system to human phenome-microRNAome network and functionally related miRNA network. Shi et al. [19] developed a computational framework to identify miRNA-disease associations by preforming random walk with restart. The method utilized the function connections between miRNAtargets and disease genes in protein-protein interaction (PPI) networks. Mrk et al. [20] presented a miRNA-protein-disease association prediction model (miRPD) in which miRNAs are linked to diseases via the underlying proteins. However, these methods largely relied on miRNA-target interactions which have high false-positive rate and false-negative rate, so they cannot achieve satisfying prediction performance.

To solve the above-mentioned problem, some similarity-based methods without relying on miRNA-target interactions were proposed. Similarity computation strategy is the key issue for miRNA-disease prediction [21]. Xuan et al. [22] presented a prediction algorithm called HDMP. HDMP predicts potential disease-associated miRNAs based on weighted *k*-nearest similar neighbors. However, HDMP could not predict miRNAs (diseases) associated with new diseases (miRNAs) due to local similarity networks. So, some global similarity-based methods were proposed, which construct a heterogeneous global network by integrating miRNA similarity, disease similarity, and known human miRNA-disease associations. For example, Chen et al. [23] proposed a global network-based prediction model, RWRMDA, to infer potential miRNA-disease association by implementing the random walk algorithm on a global network. However, it was not applicable for new diseases without any known associated miRNAs. Xuan et al. [24] proposed another prediction model called MIDP based on random walk. Compared with RWRMDA, it could predict related miRNA for new diseases. Liu et al. [25] proposed a miRNA-disease association prediction method by random walk on heterogeneous network constructed by integrating multiple data sources. In addition, some improved algorithms based on random walk were proposed [26, 27]. In addition to the random walk algorithm, other global network-based methods were proposed. For example, Chen et al. [28] developed the model for miRNA-disease association prediction (WBSMDA) by utilizing within score and between score. The within-score can capture miRNA similarity and disease similarity in known miRNA disease pairs, and the between-score can capture miRNA similarity and disease similarity in unknown miRNA-disease pairs. Next year, Chen et al. [29] proposed a computational model based on super-disease and miRNA for potential miRNA-disease association (SDMMDA) prediction. You et al. [30] proposed a path-based miRNA-disease association (PBMDA) prediction model. PBMDA adopted depth-first search algorithm on a heterogeneous graph. Zeng et al. [31] applied link prediction algorithm named structural perturbation method (SPM) on the miRNA-disease bilayer network to predict potential miRNA-disease associations. Chen et al. [32] proposed a computational model of bipartite network projection for miRNA-disease association (BNPMDA) prediction. The model took advantage of the agglomerative hierarchical clustering and improved the baseline algorithm of bipartite network recommendation based on the constructed bias ratings. In addition, some researcher utilized lncRNA-related other information to predict potential miRNA-disease associations. Chen et al. [33] developed a triple layer heterogeneous network miRNA-disease association (TLHNMDA) prediction model. In the model, the triple layer network was constructed by integrating the known miRNA-disease associations, miRNA-lncRNA interactions, miRNA function similarity, disease semantic similarity, and Gaussian interaction profile kernel similarity. Zhao et al. [34] developed a computational method based on a distance correlation set to predict miRNA-disease associations (DCSMDA), which integrated known lncRNA-disease associations, known miRNA-lncRNA associations, disease semantic similarity, and various lncRNA and disease similarity measures to construct a miRNA-lncRNA-disease network.

Furthermore, many computational models using machine learning to identify potential associations between miRNAs and diseases have also begun to appear. Chen and Yan [35] proposed regularized least squares for miRNA-disease association (RLSMDA) to uncover the relationship between diseases and miRNAs. Next, Chen and Huang [36] presented a prediction model based on Laplacian regularized sparse subspace learning called LRSSLMDA, which extracted two informative feature profiles by performing feature extraction from the integrated similarity. Chen et al. [37] developed a model of random forest for miRNA-disease association (RFMDA) prediction based on machine learning. Liang et al. [38] developed a method to discover disease-related candidate miRNAs based on adaptive multiview multilabel learning. Zhao et al. [39] developed adaptive boosting for miRNA-disease association (ABMDA) prediction to predict potential associations between diseases and miRNAs, which can balance the positive and negative samples by performing random sampling based on *k*-mean clustering on negative samples, and integrated weak classifiers to form a strong classifier based on corresponding weights. Wang et al. [40] proposed miRNA-disease association prediction model- (LMTRDA) based logistic model tree, which fused multisource information, especially the introduced miRNA sequence information. Chen et al. [41] proposed an ensemble of decision tree-based miRNA-disease association (EDTMDA) prediction model, which is a computational framework of integrating ensemble learning and dimensionality reduction. Deep learning can capture hidden, complex, and nonlinear relationships from the original data. Deep learning has been applied to various fields of bioinformatics. With the rapid development of deep learning, some deep learning-based methods have been proposed to solve the problem about miRNA-disease association prediction. For example, Chen et al. [42] proposed a model of restricted Boltzmann machine to predict multiple types of miRNA-disease association (RBMMMDA). Xuan et al. [43] presented the convolutional network-based methods for predicting candidate disease. Zeng et al. [44] developed a neural network model to predict miRNA-disease associations (NNMDA). NNMDA not only aggregated the neighbor information during the process but also preserved the topology of the original network at the same time. Gong et al. [45] proposed a network embedding-based multiple information integration method (NEMII) for miRNA-disease association prediction. Peng [46] proposed a learning-based framework, MDA-CNN, for miRNA-disease association identification. The model captures interaction features based on disease similarity network, miRNA similarity network, and protein-protein interaction network and employed an autoencoder to identify the essential feature combination for each miRNA-disease pair, and it used a convolutional neural network to predict the final label. Chen et al. [47] developed a model of deep-belief network for miRNA-disease association (DBNMDA) prediction. DBNMDA utilizes the information of all miRNA-disease pairs by introducing the unsupervised pretraining process. Then, according to the parameters obtained by pretraining, positive samples and the same number of randomly selected negative samples were applied to fine-tune deep-belief network.

Recently, some researchers have introduced the recommendation system to predict miRNA-disease association. Matrix factorizations are widely used in the recommendation systems. Some computational models based on matrix completion have been proposed. For example, Li et al. [48] proposed a miRNA-disease association prediction method based on matrix completion (MCMDA). MCMDA could not predict miRNAs for new diseases with no associations. In order to solve this problem, Chen et al. [49] proposed a computational model-based inductive matrix completion for miRNA-disease association prediction (IMCMDA). The model integrated miRNA functional similarity, disease semantic similarity, and Gaussian interaction profile kernel similarity. In addition, Chen et al. [50] integrated neighborhood constraint with matrix completion and proposed a computational model based neighborhood constraint matrix completion for miRNA-disease association (NCMCMDA) prediction. On the other hand, matrix decomposition is also used for identifying potential miRNA-disease associations. For example, Xiao et al. [51] proposed a prediction framework called graph regularized nonnegative matrix factorization (GRNMF) to infer the unknown miRNA-disease associations in heterogeneous omics data. Chen et al. [52] took advantage of the matrix factorization and network algorithm to develop a matrix decomposition and heterogeneous graph inference (MDHGI) for miRNA-disease association prediction. Cui et al. [53] proposed a robust collaborative matrix factorization method to predict novel miRNA-disease associations. The method improved the prediction accuracy by introducing the weighted *K* nearest known neighbors and the *L*_2,1_‐norm. Gao et al. [54] presented a computational framework based on graph Laplacian regularized L_2,1_-nonnegative matrix factorization (GRL_2,1_‐NMF) for inferring possible disease-connected miRNAs.

To further improve the prediction performance, we study to predict potential miRNA-disease associations based on matrix factorization and deep learning. We propose a new miRNA-disease association prediction method called DCFMDA, which combines the multilayer perceptron (MLP) and the neural nonnegative matrix factorization (NNMF) in a deep collaborative filtering framework. Firstly, we obtain miRNA and disease similarity matrices by integrating multiple heterogeneous data. Then, we utilize MLP to extract high-level features from miRNA and disease similarity matrices and decompose the known miRNA-disease association into two low rank matrices by NNMF. Finally, we merge the output of the MLP submodel and the NNMF submodel to obtain prediction results for miRNA-disease potential associations.

The rest of this paper is organized as follows.  Section 2 describes the data and method.  Section 3 presents experimental results.  Section 4 summarizes the paper.

## 2. Data and Methods

### 2.1. Data

#### 2.1.1. Human miRNA-Disease Association

HMDD is a database that curated experiment-supported evidence for human miRNA-disease associations. We downloaded known miRNA-disease association data from HMDD V2.0 [55], which includes 5430 experimentally verified miRNA-disease associations between 383 miRNAs and 495 diseases. We used adjacency matrix *A* ∈ ℝ^*M*×*N*^ to formalize the miRNA-disease associations, where *M* and *N* are the number of miRNAs and diseases, respectively. If miRNA *m* is experimentally verified to be related with disease *d*, the value of *A*(*m*, *d*) is 1, otherwise 0.

#### 2.1.2. Disease Semantic Similarity

In the National Library of Medicine MeSH, each disease is described as a hierarchical Directed Acyclic Graph (DAG). As described in [56], the disease semantic similarity can be calculated based on these DAGs. For example, disease *d* can be represented as a graph DAG(*d*) = (*d*, *T*_*d*_, *E*_*d*_), where *T*_*d*_ is the disease set of all ancestor nodes of disease *d* including disease *d* itself and *E*_*d*_ is the edge set of corresponding links. The contribution of disease *t* in DAG to the semantic value of disease *d* is defined as follows:

(1) $$\begin{array}{c} \left\{\begin{array}{cc} D_{d}\left(t\right)=1, & \text{if }t=d,\\ D_{d}\left(t\right)=\max\left\{\frac{1}{2}D_{d}\left(t^{\prime }\right)\;|\;\;t^{\prime }\in C\right\}, & \text{if }t\neq d, \end{array}\right. \end{array}$$

where *C* is children set of *t*.

The semantic value DD(*d*) of disease *d* is calculated by

(2) $$\begin{array}{c} \text{DD}\left(d\right)=\underset{t\in T_{d}}{\sum }D_{d}\left(t\right). \end{array}$$

The larger the part the two diseases share in their DAGs, the higher the similarity between the two diseases is. The semantic similarity SSD(*d*_*i*_, *d*_*j*_) of disease *d*_*i*_ and *d*_*j*_ is defined as follows:

(3) $$\begin{array}{c} \text{SSD}\left(d_{i},d_{j}\right)=\frac{\sum_{t\in T_{d_{i}}\cap T_{d_{j}}}\left(D_{d_{i}}\left(t\right)+D_{d_{j}}\left(t\right)\right)}{DD\left(d_{i}\right)+DD\left(d_{j}\right)},\;i,j=\left\{1,2,\cdots ,N\right\}. \end{array}$$

#### 2.1.3. Disease Functional Similarity by Functional Gene Network

The score of functional similarity between two diseases can be measured by disease-gene and gene-gene association data [57]. The functional similarity FSD(*g*_*i*_, *g*_*j*_) between gene *g*_*i*_ and *g*_*j*_ is defined as follows:

(4) $$\begin{array}{c} \text{FSD}\left(g_{i},g_{j}\right)=\left\{\begin{array}{cc} 1, & i=j,\\ {\text{LLS}}_{N}\left(g_{i},g_{j}\right), & i\neq j,\;e\;\left(i,j\right)\in E\left(H\right),\\ 0, & i\neq j,\;e\left(i,j\right)\notin E\left(H\right), \end{array}\right. \end{array}$$

where *e*(*i*, *j*) denotes edge between gene *g*_*i*_ and *g*_*j*_, *E*(*H*) denotes the set of all edges in the HumanNet V2 database [58], and LLS_*N*_(*g*_*i*_, *g*_*j*_) is an associated log likelihood score (LLS) that measures the probability of a functional linkage between gene *g*_*i*_ and *g*_*j*_ after normalization.

The functional association *F*_*G*_(*g*) between gene *g* and gene set *G* = {*g*_1_, *g*_2_, ⋯, *g*_*k*_} is defined as follows:

(5) $$\begin{array}{c} F_{G}\left(g\right)=\underset{1\leq i\leq k}{\max}\left(\text{FSD}\left(g,g_{i}\right)\right), \end{array}$$

where *k* indicates the number of genes in *G*, *g*_*i*_ is the *i*th gene of *G*, *i* = 1, 2, ⋯, *k*.

The functional similarity FSD(*d*_*i*_, *d*_*j*_) between disease *d*_*i*_ and *d*_*j*_ is defined as follows:

(6) $$\begin{array}{c} \text{FSD}\left(d_{i},d_{j}\right)=\text{FSD}\left(G_{1},G_{2}\right)=\frac{\sum_{1\leq i\leq m}F_{G_{2}}\left(g_{1i}\right)+\sum_{1\leq j\leq n}F_{G_{1}}\left(g_{2j}\right)}{m+n}, \end{array}$$

where *G*_1_ = {*g*_11_, *g*_12_, ⋯, *g*_1*m*_} and *G*_2_ = {*g*_21_, *g*_22_, ⋯, *g*_2*n*_} are gene set related to diseases *d*_*i*_ and *d*_*j*_, respectively, *m* is the number of genes in *G*_1_, and *n* is the number of genes in *G*_2_.

#### 2.1.4. miRNA Functional Similarity

We obtained the miRNA functional similarity data by the method provided in [56]. In the previous subsection, we have described calculating the semantic similarity between diseases. The functional similarity for each miRNA pair was calculated based on the semantic similarity of diseases. Firstly, the similarity between disease *d* and disease group *D* = {*d*_1_, *d*_2_, ⋯, *d*_*k*_} is calculated by

(7) $$\begin{array}{c} S\left(d,D\right)=\underset{1\leq i\leq k}{\max}\left(\text{SSD}\left(d,d_{i}\right)\right), \end{array}$$

where *k* denotes the number of diseases in *D* and *d*_*i*_ is the *i*th disease of *D*, *i* = 1, 2, ⋯, *k*.

Then, calculation of the functional similarity between miRNA *m*_*i*_ and *m*_*j*_ is equal to calculating the similarity between *D*1 and *D*2, where *D*1 and *D*2 represent the related disease sets of miRNA *m*_*i*_ and *m*_*j*_, respectively. Finally, the matrix FSM_*M*×*M*_ is used to denote the miRNA functional similarity. FSM(*m*_*i*_, *m*_*j*_) represents the functional similarity between miRNAs *m*_*i*_ and *m*_*j*_, which is calculated as follows:

(8) $$\begin{array}{c} \text{FSM}\left(m_{i},m_{j}\right)=\frac{\sum_{1\leq s\leq \left|D2\right|}S\left(d_{s},D1\right)+\sum_{1\leq t\leq \left|D1\right|}S\left(d_{t},D2\right)}{\left|D1\right|+\left|D2\right|}. \end{array}$$

#### 2.1.5. Gaussian Interaction Profile Kernel Similarity

Gaussian kernel is a commonly used kernel function, which has been proven effective for measuring both miRNA similarity and disease similarity [59]. The interaction profile IP(*d*_*i*_) of disease *d*_*i*_ is the *i*th column vector of the miRNA-disease association matrix. It is a binary vector representing the presence or absence of its associations with each miRNA. Gaussian interaction profile kernel similarity GD(*d*_*i*_, *d*_*j*_) between disease *d*_*i*_ and *d*_*j*_ is defined as follows [59]:

(9) $$\begin{array}{c} \text{GD}\left(d_{i},d_{j}\right)=\exp\left(-\beta_{d}{\left\|\text{IP}\left(d_{i}\right)-\text{IP}\left(d_{j}\right)\right\|}^{2}\right),\\ \beta_{d}=\frac{\beta_{d}^{\prime }}{\left(1/N\right)\sum_{i=1}^{N}{\left\|IP\left(d_{i}\right)\right\|}^{2}}, \end{array}$$

where *i*, *j* = 1, 2, ⋯, *N* and *β*_*d*_ is used to control kernel bandwidth, which is obtained by normalizing the average number  *β*_*d*_′ of associated miRNAs per disease.

Similarly, the Gaussian interaction profile kernel similarity GM(*m*_*i*_, *m*_*j*_) between miRNA *m*_*i*_ and *m*_*j*_ is defined as follows:

(10) $$\begin{array}{c} \text{GM}\left(m_{i},m_{j}\right)=\exp\left(-\beta_{m}\right.{\left\|\text{IP}\left(m_{i}\right)-\text{IP}\left(m_{j}\right)\right\|}^{2},\\ \beta_{m}=\frac{\beta_{m}^{\prime }}{\left(1/M\right)\sum_{i=1}^{M}{\left\|IP\left(m_{i}\right)\right\|}^{2}}, \end{array}$$

where *i*, *j* = 1, 2, ⋯, *M*.

#### 2.1.6. Integrated Similarity for miRNAs and Diseases

In order to overcome the shortcomings of single similarity measure to accurately reflect the characteristics of miRNA similarity and disease similarity from different perspectives, some method integrated multiple different similarity measure data to construct the miRNA similarity matrix and disease similarity matrix to improve the prediction performance.

We integrate miRNA functional similarity FSM and miRNA Gaussian interaction profile kernel similarity GM to construct the miRNA similarity matrix ISM(*m*_*i*_, *m*_*j*_) between miRNA *m*_*i*_ and *m*_*j*_.

(11) $$\begin{array}{c} \text{ISM}\left(m_{i},m_{j}\right)=\left\{\begin{array}{cc} \text{FSM}\left(m_{i},m_{j}\right), & \text{if FSM}\left(m_{i},m_{j}\right)\neq 0,\\ \text{GM}\left(m_{i},m_{j}\right), & \text{otherwise}. \end{array}\right. \end{array}$$

We also integrate disease semantic similarity SSD, the disease functional similarity FSD, and the disease Gaussian interaction profile kernel similarity GD to construct the disease similarity matrix ISD(*d*_*i*_, *d*_*j*_) between disease *d*_*i*_ and *d*_*j*_. The formula is as follows:

(12) $$\begin{array}{c} \text{ISD}\left(d_{i},d_{j}\right)=\left\{\begin{array}{cc} \frac{\text{FSD}\left(d_{i},d_{j}\right)+\text{SSD}\left(d_{i},d_{j}\right)}{2}, & \text{if FSD}\left(d_{i},d_{j}\right)\neq 0\text{ or SSD}\left(d_{i},d_{j}\right)\neq 0,\\ \text{GD}\left(d_{i},d_{j}\right), & \text{ otherwise}. \end{array}\right. \end{array}$$

### 2.2. Methods

As a universal computational algorithm, the recommendation algorithm has been applied in many fields including bioinformatics. The miRNA-disease association prediction can be regarded as a recommendation problem. This kind of prediction method regards miRNAs as users and diseases as commodities and recommends miRNAs to a disease according to its known preference on miRNAs and vice versa. Traditional recommendation models are mainly divided into collaborative filtering, content-based recommendation system, and hybrid recommendation system. Recently, researchers have proposed some recommendation algorithms using deep learning to overcome the shortcomings of traditional collaborative filtering models [60].

**Figure 1:**
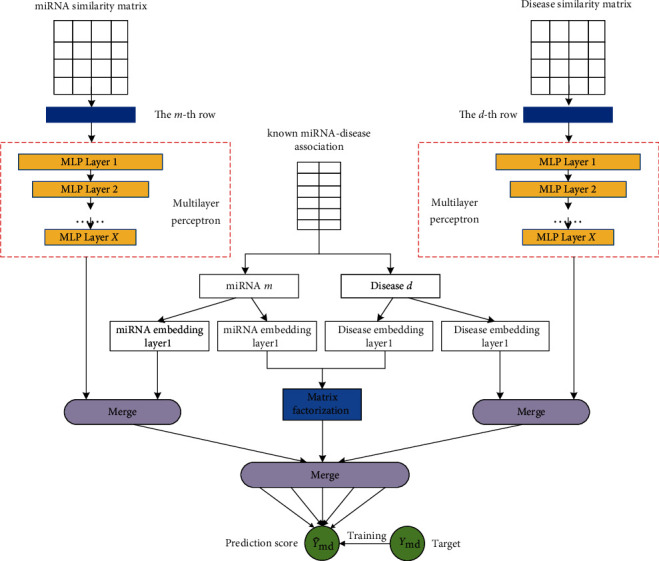
Workflow of deep collaborative filtering for miRNA-disease association prediction.

In this paper, we proposed a new deep collaborative filtering framework for miRNA-disease association prediction called DCFMDA. This method combines the multilayer perceptron (MLP) submodel and neural nonnegative matrix factorization (NNMF) submodel in deep collaborative filtering framework. Firstly, in the MLP submodel, the *m*th row of the miRNA similarity matrix (i.e., the similarity data between miRNA *m* and all the other miRNAs) was fed into a multilayer perception, and the *d*th row of the disease similarity matrix (i.e., the similarity data between disease *d* and all the other diseases) was fed into another multilayer perception. The two MLPs would be trained to learn high-level biological patterns from miRNA similarity and disease similarity, respectively. Secondly, all known miRNA-disease association pairs were fed into the neural NNMF submodel to train. Finally, the output of the two submodels was merged to get prediction scores of miRNA-disease pairs. The proposed method is shown in Figure 1.

#### 2.2.1. Neural Nonnegative Matrix Factorization (NNMF)

Matrix factorization (MF) based approaches are proven to be highly accurate and scalable in addressing collaborative filtering (CF) problems [61]. The purpose of nonnegative matrix factorization (NMF) is to find two nonnegative matrices whose product is optimal approximation to the original matrix. Given miRNA-disease association matrix A, it can be decomposed into the product of two low rank nonnegative matrices *W* and *H*, namely, *A* ≈ *WH*^*T*^. Solving the problem of prediction miRNA-disease potential associations using NMF can be described as the following objective function:

(13) $$\begin{array}{c} \min{\left\|A-WH^{T}\right\|}_{F}^{2}. \end{array}$$

However, the matrix factorization method only uses the fixed inner product to predict miRNA-disease associations, which leads to some limitation of prediction algorithm. So we use a nonnegative matrix factorization submodel (NNMF) based on a two-layer fully connected neural network to predict the potential association between miRNA and disease. In the NNMF submodel, one-hot encodings of miRNA *i* and disease *j*are used as the input vectors and two embedding vectors *m*_*i*_ and *d*_*j*_ are obtained by the embedding layer. We have two two-layer fully connected neural networks to transform the representations of *m*_*i*_ and *d*_*j*_. Through the neural network, *m*_*i*_ and *d*_*j*_ are mapped to low-dimensional vectosr *p*_*i*_ and *q*_*j*_ in a latent space, respectively. The miRNA-disease association prediction submodel based on NNMF is as follows:

(14) $$\begin{array}{c} {\hat{y}}_{ij}=f^{\text{NNMF}}\left(i,j\;|\;p_{i},q_{j}\right)=p_{i}^{T}q_{j}=p_{i}⊙q_{j}, \end{array}$$

where *i* = 1, 2, ⋯, *M*, *j* = 1, 2, ⋯, *N*

#### 2.2.2. Multilayer Perceptron (MLP)

**Figure 2:**
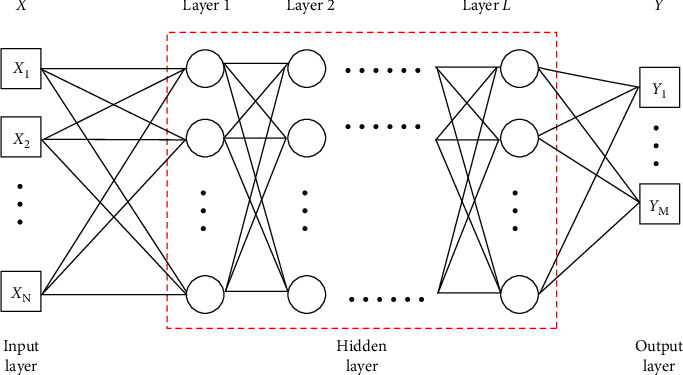
The multilayer perceptron.

Multilayer perceptron (MLP) is a deep learning structure, which is a feedforward neural network with multiple hidden layers between input and output layers. A single layer perceptron cannot classify linear inseparable problems, but the multilayer perceptron can overcome this weakness by the nonlinear mapping of input space based on activation function. MLP has high ability of nonlinear modeling. The structure of the multilayer perceptron model is shown in Figure 2.

We regard the miRNA-disease association prediction as a binary classification problem. That is, if there is a correlation between miRNA *i* and disease *j*, the corresponding label will be 1, otherwise 0. We use a submodel based on MLP to solve the problem for miRNA-disease association prediction. The number of neurons in each hidden layer of MLP is less than that in the previous layer. The number of neurons in the first hidden layer is equal to the dimension of the input vector, and the number of neurons in the last hidden layer is equal to the dimension of the output vector. Formally, we denote the input vector by *X*, the output vector by *Y*, the number of hidden layers by *L*, the connection weight matrix from hidden layer *l* − 1 to hidden layer *l* by *W*_*l*_, and the bias vector of the *l*th layer by *b*_*l*_, where 1 ≤ *l* ≤ *L*. The MLP model is formulated as follows [62]:

(15) $$\begin{array}{c} a_{0}=X,\\ a_{1}=\theta_{1}\left(W_{1}^{T}a_{0}+b_{1}\right),\\ a_{2}=\theta_{2}\left(W_{2}^{T}a_{1}+b_{2}\right),\\ a_{L}=\theta_{L}\left(W_{L}^{T}a_{L-1}+b_{L}\right),\\ Y=\phi^{\text{MLP}}\left(X\right)=a_{L}, \end{array}$$

where *a*_*l*_ denotes the output of the *l*th layer and *θ*_*l*_ denotes the activation function of the *l*th layer. We select ReLU as the activation function of each hidden layer, which can be computed by *f*(*x*) = max(0, *x*). ReLU is employed to alleviate the problem of the gradient disappearance and solve the overfitting problem of machine learning [63].

In our proposed MLP submodel, we used two MLPs to transform the representations of miRNA and disease. The miRNA similarity matrix ISM and disease similarity matrix ISD are the input of these two multilayer perceptrons. ISM_*i*_ is the *i*th row of matrix ISM, which represents the similarity feature of miRNA *m*_*i*_. ISD_*j*_ is the *j*th row of matrix ISD, which represents similarity feature of disease *d*_*j*_. We used ISM_*i*_ to train the left MLP and used ISD_*j*_ to train the right MLP. Through the neural network, ISM_*i*_ and ISD_*j*_ are finally mapped to a low-dimensional vector in a latent space. So the similarity feature vectors of the miRNA *m*_*i*_ and disease *d*_*j*_ can be formulated as follows:

(16) $$\begin{array}{c} m_{i}^{\text{MLP}}=\phi^{\text{MLP}}\left({\text{ISM}}_{i}\right),\\ d_{j}^{\text{MLP}}=\phi^{\text{MLP}}\left({\text{ISD}}_{j}\right). \end{array}$$

The miRNA-disease association prediction submodel based on MLP is as follows:

(17) $$\begin{array}{c} {\hat{y}}_{ij}=f^{\text{MLP}}\left({\text{ISM}}_{i},{\text{ISD}}_{j}\;|\;m_{i}^{\text{MLP}},d_{j}^{\text{MLP}}\right)=\sigma \left(m_{i}^{\text{MLP}},d_{j}^{\text{MLP}}\right), \end{array}$$

where *i* = 1, 2, ⋯, *M*, *j* = 1, 2, ⋯, *N*, *σ*(*x*) = 1/(1 + *e*^−*x*^).

#### 2.2.3. Method DCFMDA

We construct a prediction model based on NNMF and MLP. We capture the linear relationship between miRNAs and diseases by NNMF and learn the nonlinear relationship between miRNAs and diseases by MLP. The NNMF submodel and the MLP submodel share the embedding layer.

The NNMF submodel learns from known miRNA-disease association to obtain the original prediction score. The MLP submodel learns the low-dimensional feature of miRNA and disease from the miRNA similarity matrix and disease similarity matrix, respectively. Finally, we merge outputs of the NNMF submodel and MLP submodel to obtain the final prediction score for disease-related miRNAs. The presented model is formulated as follows:

(18) $$\begin{array}{c} y_{ij}^{\text{NNMF}}=f^{\text{NNMF}}\left(i,j\;|\;m_{i}^{\text{NNMF}},d_{j}^{\text{NNMF}}\right)=m_{i}^{\text{NNMF}}⨀d_{j}^{\text{NNMF}}, \end{array}$$

(19) $$\begin{array}{c} m_{i}^{\text{MLP}}=\phi^{\text{MLP}}\left({\text{ISM}}_{i}\right), \end{array}$$

(20) $$\begin{array}{c} d_{j}^{\text{MLP}}=\phi^{\text{MLP}}\left({\text{ISD}}_{j}\right), \end{array}$$

(21) $$\begin{array}{c} m_{i}^{\text{DCFMDA}}=\left[\begin{array}{c} m_{i}^{\text{MLP}}\\ m_{i} \end{array}\right], \end{array}$$

(22) $$\begin{array}{c} d_{j}^{\text{DCFMDA}}=\left[\begin{array}{c} d_{j}^{\text{MLP}}\\ d_{j} \end{array}\right], \end{array}$$

(23) $$\begin{array}{c} {\hat{y}}_{ij}=\sigma \left(\left[\begin{array}{c} y_{ij}^{\text{NNMF}}\\ m_{i}^{\text{DCFMDA}}\\ d_{j}^{\text{DCFMDA}} \end{array}\right]\right), \end{array}$$

where *m*_*i*_ and *d*_*j*_ are two embedding vectors of miRNA *i* and disease *j*, respectively. ISM_*i*_ is the similarity feature of miRNA *i*, ISD_*j*_ is the similarity feature of disease *j*, *m*_*i*_^MLP^ denotes the output of the left MLP, *d*_*j*_^MLP^ denotes the output of the right MLP, *y*_*ij*_^NNMF^ denotes original prediction score, *m*_*i*_^DCFMDA^ is obtained by concatenating *m*_*i*_^MLP^ with the miRNA embedding vector *m*_*i*_, and *d*_*j*_^DCFMDA^ is obtained by concatenating *d*_*j*_^MLP^ with the disease embedding vector *d*_*j*_.

The final layer of the DCFMDA is used for the classification task, its activation function *σ*(*x*)is the sigmoid function *σ*(*x*) = 1/(1 + *e*^−*x*^).

In our proposed model, we use the binary crossentropy loss function *ℒ*:

(24) $$\begin{array}{c} L=-\underset{\left(i,j\right)\in A}{\sum }y_{ij}\log\;{\hat{y}}_{ij}+\left(1-y_{ij}\right)\log\left(1-{\hat{y}}_{ij}\right), \end{array}$$

where (*i*, *j*) ∈ *A* is the index of the training examples, *y*_*ij*_ represents the true label for the given input sample (*i*, *j*), and ${\hat{y}}_{ij}$ represents predicted result. The purpose of deep learning is to minimize loss function through continuous training iterations to get the best prediction.

Algorithm 1 describes our proposed miRNA-disease association prediction algorithm using deep collaborative filtering called DCFMDA.

## 3. Result

### 3.1. Performance Evaluation

To evaluate the prediction performance of algorithm DCFMDA, we perform a 10-fold cross-validation on known experimentally verified miRNA-disease associations. The 5430 experiment-supported miRNA-disease associations are considered as positive samples. We are not sure which miRNAs are not associated with diseases. So, for each known miRNA-disease pair, we will randomly sample four unobserved miRNA-disease pairs as negative samples. For the 10-fold cross-validation, all positive and negative samples are randomly divided into ten parts. In each fold, nine of the ten parts are used for the training model in turn, and the remaining one is used as test samples.

**Figure 3:**
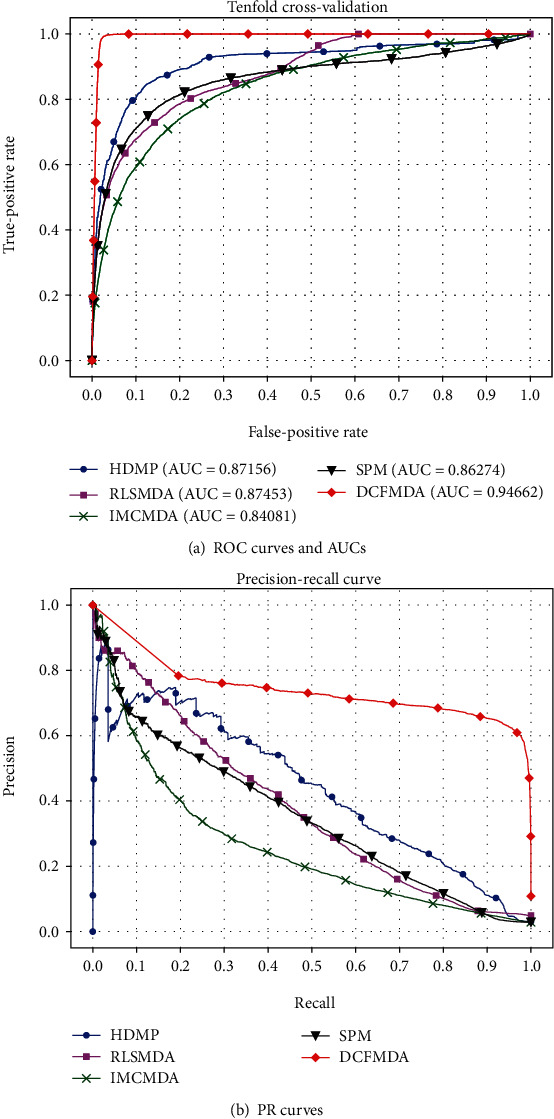
Performance comparison for HDMP, RLSMDA, IMCMDA, SPM, and DCFMDA in terms of ROC curve, AUC, and PR curve.

We use the receiver operating characteristic (ROC) curve, area under ROC (AUC), precision-recall (PR) curve, and F1 score to evaluate the performance of the predictive algorithm. The ROC curve plots the true-positive rate (TPR) versus the false-positive rate (FPR) at different thresholds. The value of AUC is usually between 0.5 and 1. When AUC is 1, it means that the prediction result will achieve the best effect. Our data is seriously unbalanced, because the number of negative samples (unconfirmed miRNA-disease associations) is much larger than the number of positive samples (experiment-supported miRNA-disease associations). Therefore, we also draw a PR curve to evaluate the prediction ability of different miRNA-disease association prediction algorithms. We compare DCFMDA with four existing miRNA-disease prediction algorithms HDMP, RLSMDA, IMCMDA, and SPM.  Figure 3 shows ROC curves and PR curves of the five prediction algorithms and reports their corresponding AUCs in a 10-fold cross-validation on experimentally verified miRNA-disease associations.  Figure 3(a) shows that DCFMDA almost always has the highest TPRs under the same false-negative rates, and obtains the highest AUC (0.94662) among these algorithms, whereas the AUCs of HDMP, RLSMDA, IMCMDA, and SPM are 0.87156, 0.87453, 0.84081, and 0.86274, respectively.  Figure 3(b) shows that DCFMDA achieves a higher precision than all the other algorithms for any given recall value. For ROC curve or PR curve, DCFMDA performs significantly better than the other four algorithms. F1 score is the harmonic mean of both metrics of recall and precision. Since there is a trade-off between precision and recall, F1 score is also used to evaluate the performance of algorithms.  Table 1 shows the F1 score of the top-*K* candidates. The F1 score of DCFMDA is more stable, while F1 scores of the other four algorithms were decreasing from the top 50 to top 1000. From Table 1, we can see that DCFMDA achieved better performance than other algorithms in terms of the F1 score.

The experiment results indicate that our method can achieve higher prediction performance. One reason is that we introduce deep learning into miRNA-disease prediction and effectively integrated neural nonnegative matrix factorization and multilayer perceptron technology to predict potential miRNA-disease associations. Another reason is that different miRNA similarity and disease similarity data are used to construct the miRNA similarity matrix and the disease similarity matrix.

### 3.2. Case Study

In order to further verify the prediction performance of DCFMDA, we carried out case studies on five diseases including breast neoplasms, colon neoplasms, kidney neoplasms, leukemia, and lymphoma. From HMDD V2.0, we obtained 5430 known associations and 184155 unknown associations between 495 miRNAs and 383 diseases. For our case study, all the known miRNA-disease associations were used as training samples, and other unknown associations were regarded as candidate associations for validation. For each investigated disease, we ranked candidate miRNAs according to their predicted scores and selected the top-50 candidate miRNAs to verify whether the candidate miRNAs were associated with the current disease by two other databases, namely, dbDEMC [64] and miRCancer [65], as well as published literatures. The validation results are shown in Table 2. The database dbDEMC 2.0 is an integrated database that documents 209 expression profiling data sets with 36 cancer types and 73 subtypes, and a total of 2224 differentially expressed miRNAs were identified. It allows users to make a quick search of the differentially expressed miRNAs in certain cancer types. The database miRCancer is a miRNA-cancer association database that provides comprehensive collection of miRNA expression profiles in various human cancers. A user can search the database by miRNA or cancer name.

There are intersections between known miRNA-disease associations obtained from databases HMDD V2.0, dbDEMC, and miRCancer. For example, 546 of the 5430 known miRNA-disease associations in HMDD V2.0 also exist in dbDEMC 2.0. Because we only predict and verify the candidate miRNAs unrelated to the investigated disease in HMDD V2.0, none of these candidate miRNAs exist in HMDD V2.0. We can be sure that the validation of candidate miRNAs is completely independent of HMDD V2.0.

Breast neoplasms are one of the most common cancers for women. We have inferred associations between all the candidate miRNAs for breast neoplasm and confirmed 49 of the top-50 candidate miRNAs to be association with breast cancer by dbDEMC, miRCancer, and published literatures (see Table 3). Because the same miRNA gene may have different identifiers, we can use the alias to verify whether the miRNA is associated with breast cancer. We obtain alias of miRNAs by retrieving the miRBase and GeneCards databases. For example, hsa-mir-371 (the alias of hsa-mir-371a) and has-mir-642 (the alias of hsa-mir-642) can be confirmed to be related to breast cancer by dbDEMC2.

Colon neoplasms are a common malignant tumor of the digestive tract occurring in the colon. Various evidences indicate that miRNAs potentially play an important role in predicting markers of early diagnosis, prognosis, and chemosensitivity of colon cancer [66]. DCFMDA has inferred associations between all the candidate miRNAs for colon cancer, and all top-50 candidate miRNAs are confirmed to be associated with colon neoplasms by dbDEMC, miRCancer, and published literatures (see Table 4).

Kidney neoplasms are one of the most rapidly growing malignant tumors. Abnormal expression of miRNAs has been detected in several kinds of kidney cancers. For example, compared with the normal samples, the expression of hsa-mir-194 (the third in Table 5) was reported to be downregulated in kidney neoplasm patients. Literature [67] confirmed that the expression level of hsa-mir-378 (the twelfth in Table 5) is up-regulated in the blood of patients with renal cell carcinoma compared to healthy controls. Predicting miRNAs related with kidney neoplasm by DCFMDA, 49 of the top-50 candidate miRNAs have been validated (see Table 5). Considering the different names of the same miRNA, we can find previous IDs of these miRNAs in the miRBase database. For example, hsa-mir-378a cannot be retrieved to be related with kidney cancer from database dbDEMC and miRCancer, but it can be retrieved by its previous ID has-mir-378.

Leukemia is a cancer caused by an overproduction of damaged white blood cells. It is the most common cancer among people under 15 years old. MiRNAs play an important role in the development of leukemia. One of the most typical examples is the association of miR-15a and miR-16a with chronic lymphocytic leukemia. Researchers found that 65% of B cell chronic lymphoblastic leukemia patients have deletions of chromosome 13q14, a locus that includes miR-15a and miR-16a, which consequently present downregulated expression [10]. In our case study, 46 of the 50 candidate miRNAs related to leukemia have been verified by relevant databases (see Table 6). We have verified hsa-mir-323 to be associated with leukemia by database dbDEMC, where hsa-mir-323 is the previous ID of hsa-mir-323a. We are not sure whether the remaining four of the top-50 miRNAs, namely, hsa-mir-302b, hsa-mir-216b, hsa-mir-668, and has-mir-489, are related to leukemia.

Lymphomas are the most common ones of hematologic tumors. For the top-50 lymphoma-associated miRNAs predicted by DCFMDA, 49 of them have experimental literature evidence (see Table 7). For example, Literature [68] found that the expression of hsa-mir-223 was downregulated more than twice in diffuse large B-cell lymphoma (DLBCL) .

## 4. Conclusion

Predicting disease-related miRNAs will help people understand the underlying pathogenesis of diseases. To overcome the time-consuming and expensive shortcomings of experimental methods, researchers have focused on identifying miRNA-disease potential association by computational methods. Compared with existing methods, the main contribution of our work is to propose a method of predicting potential miRNA-disease association by deep collaborative filtering. In addition to the experimental confirmed miRNA-disease association, our method constructs the miRNA similarity matrix by integrating the miRNA functional similarity and miRNA Gaussian interaction profile kernel similarity and constructs the disease similarity matrix by integrating the disease semantic similarity, disease functional similarity, and disease Gaussian interaction profile kernel similarity. The performance of our method is validated by 10-fold cross-validation and case studies. The experiment results indicate that our method can achieve effective and reliable prediction results. In the future, we will further improve the prediction performance of DCFMDA by the following three aspects. Firstly, considering that the random selection of negative samples may lead to a false negative, we will use an unsupervised deep learning model for prediction. Secondly, our model simply combined the lncRNA similarity and disease similarity as the feature vector of miRNA-disease association, which cannot accurately describe the association features. Lastly, to better integrate various miRNA similarity and disease similarity by weighted average, we will study an optimal weighting strategy so that object similarity matrices can be appropriately constructed.

## Figures and Tables

**Algorithm 1:**
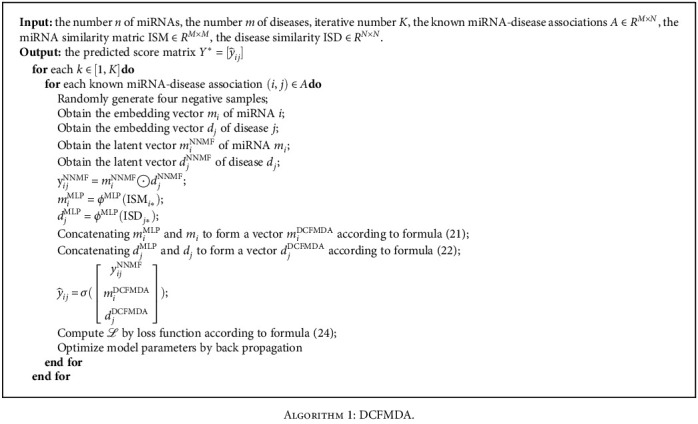
DCFMDA.

**Table 1:** Performance comparison for HDMP, RLSMDA, IMCMDA, SPM, and DCFMDA in terms of F1 score.

	Top 100	Top 200	Top 300	Top 400	Top 500	Top 600	Top 700	Top 800	Top 900	Top 1000
DCFMDA	0.8827	0.8919	0.9091	0.8996	0.8938	0.8806	0.8773	0.8803	0.8854	0.8777
HDMP	0.9130	0.9417	0.8047	0.7635	0.7760	0.8201	0.8215	0.8405	0.8424	0.8317
RLSMDA	0.9473	0.9247	0.9209	0.9262	0.9142	0.9041	0.8906	0.8788	0.8701	0.8623
IMCMDA	0.9847	0.9446	0.8785	0.8506	0.8372	0.8048	0.7784	0.7635	0.7439	0.7318
SPM	0.9583	0.9333	0.9150	0.8732	0.8345	0.8142	0.8003	0.7943	0.7879	0.7812

**Table 2:** Proportion of verified associations in the top-50 candidate miRNAs for five different diseases.

	Breast neoplasms	Colon neoplasms	Kidney neoplasms	Leukemia	Lymphoma
Percentage	98%	100%	98%	92%	98%

**Table 3:** The top-50 predicted miRNAs associated with breast neoplasms.

Rank	miRNA	Evidence	Rank	miRNA	Evidence
1	has-mir-106a	dbDEMC	26	has-mir-372	dbDEMC
2	has-mir-192	dbDEMC	27	has-mir-181d	dbDEMC
3	has-mir-449a	dbDEMC	28	has-mir-196b	dbDEMC
4	has-mir-449b	dbDEMC	29	has-mir-532	dbDEMC
5	has-mir-99b	dbDEMC	30	has-mir-198	dbDEMC
6	has-mir-483	miRCancer	31	has-mir-370	dbDEMC
7	has-mir-15b	dbDEMC	32	has-mir-513b	dbDEMC
8	has-mir-376a	dbDEMC	33	has-mir-433	dbDEMC
9	has-mir-424	dbDEMC	34	has-mir-513c	dbDEMC
10	has-mir-491	PMID:25725194	35	has-mir-362	dbDEMC
11	has-mir-144	dbDEMC	36	has-mir-615	dbDEMC
12	has-mir-181c	dbDEMC	37	has-mir-98	dbDEMC
13	has-mir-30e	miRCancer	38	has-mir-363	dbDEMC
14	has-mir-498	dbDEMC	39	has-mir-325	dbDEMC
15	has-mir-138	dbDEMC	40	has-mir-509	PMID:25659578
16	has-mir-142	PMID:25406066	41	has-mir-130b	dbDEMC
17	has-mir-371a	dbDEMC	42	has-mir-154	dbDEMC
18	has-mir-92b	dbDEMC	43	has-mir-675	dbDEMC
19	has-mir-184	dbDEMC	44	has-mir-642a	dbDEMC
20	has-mir-542	PMID:24846313	45	has-mir-500a	dbDEMC
21	has-mir-134	dbDEMC	46	has-mir-548c	PMID:25802200
22	has-mir-571	dbDEMC	47	has-mir-331	PMID:25883093
23	has-mir-185	dbDEMC	48	has-mir-381	dbDEMC
24	has-mir-32	dbDEMC	49	has-mir-519b	Unconfirmed
25	has-mir-130a	dbDEMC	50	has-mir-502	PMID:27080302

**Table 4:** The top-50 predicted miRNAs associated with colon neoplasms.

Rank	miRNA	Evidence	Rank	miRNA	Evidence
1	has-mir-30e	dbDEMC	26	has-mir-340	PMID:24448820
2	has-mir-15b	dbDEMC	27	has-mir-20a	dbDEMC
3	has-mir-193b	dbDEMC	28	has-mir-625	dbDEMC
4	has-mir-373	miRCancer	29	has-mir-486	dbDEMC
5	has-mir-16	PMID:25623762	30	has-mir-370	dbDEMC
6	has-mir-203	dbDEMC	31	has-mir-194	dbDEMC
7	has-mir-192	dbDEMC	32	has-mir-383	dbDEMC
8	has-mir-148a	dbDEMC	33	has-mir-146a	dbDEMC
9	has-mir-204	dbDEMC	34	has-mir-30b	dbDEMC
10	has-mir-106b	dbDEMC	35	has-mir-92a	dbDEMC
11	has-mir-376b	dbDEMC	36	has-mir-223	dbDEMC
12	has-mir-124	dbDEMC	37	has-mir-23b	dbDEMC
13	has-mir-122	dbDEMC	38	has-mir-32	dbDEMC
14	has-mir-132	dbDEMC	39	has-mir-497	dbDEMC
15	has-mir-143	dbDEMC	40	has-mir-93	dbDEMC
16	has-mir-10b	dbDEMC	41	has-mir-19a	dbDEMC
17	has-mir-186	dbDEMC	42	has-mir-34a	dbDEMC
18	has-mir-182	dbDEMC	43	has-mir-214	dbDEMC
19	has-mir-429	dbDEMC	44	has-mir-190a	dbDEMC
20	has-mir-125b	dbDEMC	45	has-mir-107	dbDEMC
21	has-mir-18a	dbDEMC	46	has-mir-15a	dbDEMC
22	has-mir-372	dbDEMC	47	has-mir-27a	dbDEMC
23	has-mir-96	dbDEMC	48	has-mir-31	dbDEMC
24	has-mir-212	dbDEMC	49	has-mir-424	dbDEMC
25	has-mir-19b	dbDEMC	50	has-mir-125a	dbDEMC

**Table 5:** The top-50 predicted miRNAs associated with kidney neoplasms.

Rank	miRNA	Evidence	Rank	miRNA	Evidence
1	has-mir-494	dbDEMC	26	has-mir-184	dbDEMC
2	has-mir-130b	dbDEMC	27	has-mir-122	dbDEMC
3	has-mir-194	dbDEMC	28	has-mir-15b	dbDEMC
4	has-mir-384	dbDEMC	29	has-mir-188	dbDEMC
5	has-mir-24	dbDEMC	30	has-mir-136	dbDEMC
6	has-mir-16	dbDEMC	31	has-mir-145	dbDEMC
7	has-mir-342	dbDEMC	32	has-mir-487a	PMID:25938468
8	has-mir-203	dbDEMC	33	has-mir-133b	dbDEMC
9	has-mir-150	dbDEMC	34	has-mir-561	dbDEMC
10	has-mir-186	dbDEMC	35	has-mir-125b	dbDEMC
11	has-mir-126	dbDEMC	36	has-mir-17	dbDEMC
12	has-mir-378a	dbDEMC	37	has-mir-429	dbDEMC
13	has-mir-92b	dbDEMC	38	has-mir-214	dbDEMC
14	has-mir-424	dbDEMC	39	has-mir-106a	dbDEMC
15	has-mir-20a	dbDEMC	40	has-mir-106b	dbDEMC
16	has-mir-200a	dbDEMC	41	has-mir-23a	dbDEMC
17	has-mir-31	dbDEMC	42	has-mir-127	dbDEMC
18	has-mir-372	dbDEMC	43	has-mir-451a	dbDEMC
19	has-mir-219	PMID:22440013	44	has-mir-423	dbDEMC
20	has-mir-200b	dbDEMC	45	has-mir-223	dbDEMC
21	has-mir-199a	dbDEMC	46	has-mir-189	Unconfirmed
22	has-mir-206	dbDEMC	47	has-mir-20b	dbDEMC
23	has-mir-138	dbDEMC	48	has-mir-143	dbDEMC
24	has-mir-373	dbDEMC	49	has-mir-132	dbDEMC
25	has-mir-205	miRCancer	50	has-mir-19a	dbDEMC

**Table 6:** The top-50 predicted miRNAs associated with leukemia.

Rank	miRNA	Evidence	Rank	miRNA	Evidence
1	has-mir-218	PMID:23022987	26	has-mir-494	dbDEMC
2	has-mir-221	dbDEMC	27	has-mir-219	dbDEMC
3	has-mir-222	dbDEMC	28	has-mir-214	PMID:25361012
4	has-mir-302b	Unconfirmed	29	has-mir-99a	dbDEMC
5	has-mir-452	PMID:29326345	30	has-mir-145	dbDEMC
6	has-mir-128	PMID:22209839	31	has-mir-181b	dbDEMC
7	has-let-7e	dbDEMC	32	has-mir-489	Unconfirmed
8	has-mir-142	dbDEMC	33	has-mir-146b	dbDEMC
9	has-mir-155	dbDEMC	34	has-mir-146a	dbDEMC
10	has-mir-197	dbDEMC	35	has-mir-127	dbDEMC
11	has-mir-22	dbDEMC	36	has-let-7i	dbDEMC
12	has-mir-148a	dbDEMC	37	has-mir-23a	dbDEMC
13	has-mir-20b	dbDEMC	38	has-mir-203	PMID:21323860
14	has-mir-182	dbDEMC	39	has-mir-181d	dbDEMC
15	has-mir-216b	Unconfirmed	40	has-mir-655	PMID:26340914
16	has-let-7a	dbDEMC	41	has-mir-106b	dbDEMC
17	has-mir-504	dbDEMC	42	has-mir-708	dbDEMC
18	has-mir-223	dbDEMC	43	has-mir-423	dbDEMC
19	has-mir-144	dbDEMC	44	has-mir-668	Unconfirmed
20	has-mir-34b	dbDEMC	45	has-mir-15b	dbDEMC
21	has-let-7d	dbDEMC	46	has-let-7c	dbDEMC
22	has-mir-224	dbDEMC	47	has-mir-129	dbDEMC
23	has-mir-520h	PMID:29768346	48	has-mir-323a	dbDEMC
24	has-mir-425	dbDEMC	49	has-mir-23b	dbDEMC
25	has-mir-126	dbDEMC	50	has-mir-542	dbDEMC

**Table 7:** The top-50 predicted miRNAs associated with lymphoma.

Rank	miRNA	Evidence	Rank	miRNA	Evidence
1	has-let-7a	dbDEMC	26	has-let-7g	dbDEMC
2	has-mir-494	dbDEMC	27	has-mir-208b	dbDEMC
3	has-mir-338	dbDEMC	28	has-mir-206	dbDEMC
4	has-let-7b	dbDEMC	29	has-mir-27b	dbDEMC
5	has-mir-93	dbDEMC	30	has-mir-132	dbDEMC
6	has-mir-141	dbDEMC	31	has-mir-223	dbDEMC
7	has-mir-518c	Unconfirmed	32	has-mir-208a	dbDEMC
8	has-mir-302c	dbDEMC	33	has-mir-9	dbDEMC
9	has-mir-302a	dbDEMC	34	has-let-7d	dbDEMC
10	has-mir-23b	dbDEMC	35	has-mir-378a	dbDEMC
11	has-mir-145	dbDEMC	36	has-mir-296	dbDEMC
12	has-mir-106b	dbDEMC	37	has-mir-96	dbDEMC
13	has-mir-99a	dbDEMC	38	has-mir-106a	dbDEMC
14	has-let-7e	dbDEMC	39	has-mir-483	dbDEMC
15	has-mir-31	dbDEMC	40	has-mir-422a	dbDEMC
16	has-mir-103a	dbDEMC	41	has-mir-125b	dbDEMC
17	has-mir-130b	dbDEMC	42	has-mir-152	dbDEMC
18	has-mir-205	dbDEMC	43	has-mir-183	dbDEMC
19	has-mir-192	dbDEMC	44	has-mir-34a	dbDEMC
20	has-mir-29a	dbDEMC	45	has-mir-33b	dbDEMC
21	has-mir-584	dbDEMC	46	has-mir-182	dbDEMC
22	has-let-7f	dbDEMC	47	has-mir-302d	dbDEMC
23	has-lef-7c	dbDEMC	48	has-mir-216b	dbDEMC
24	has-mir-424	dbDEMC	49	has-mir-137	dbDEMC
25	has-mir-219	dbDEMC	50	has-mir-375	dbDEMC

## Data Availability

Known miRNA-disease association data were taken from database HMDD 2.0 (http://www.cuilab.cn/hmdd), human microRNA functional similarity (http://www.lirmed.com/misim/), and disease semantic similarity (https://github.com/IMCMDAsourcecode/IMCMDA).
